# Ovarian carcinosarcoma is highly aggressive compared to other ovarian cancer histotypes

**DOI:** 10.3389/fonc.2024.1399979

**Published:** 2024-05-24

**Authors:** Iona McFarlane, Joanna M. Porter, Elizabeth Brownsell, Nidal Ghaoui, Kathryn C. Connolly, C. Simon Herrington, Robert L. Hollis

**Affiliations:** ^1^ The Nicola Murray Centre for Ovarian Cancer Research, Cancer Research UK Scotland Centre, Institute of Genetics and Cancer, University of Edinburgh, Edinburgh, United Kingdom; ^2^ The Simpson Centre for Reproductive Health, Royal Infirmary of Edinburgh, Edinburgh, United Kingdom; ^3^ Edinburgh Cancer Centre, Western General Hospital, NHS Lothian, Edinburgh, United Kingdom

**Keywords:** ovarian cancer, carcinosarcoma, malignant mixed mullerian tumour, survival, ovarian carcinoma

## Abstract

**Background:**

Ovarian carcinosarcoma (OCS) is an unusual ovarian cancer type characterized by distinct carcinomatous and sarcomatous components. OCS has been excluded from many of the pan-histotype studies of ovarian carcinoma, limiting our understanding of its behavior.

**Methods:**

We performed a multi-cohort cross-sectional study of characteristics and outcomes in ovarian cancer patients from Scotland (n=2082) and the Surveillance, Epidemiology and End Results Program (SEER, n=44946) diagnosed with OCS or one of the other major histotypes: high grade serous (HGSOC), endometrioid (EnOC), clear cell (CCOC), mucinous (MOC) or low grade serous ovarian carcinoma (LGSOC). Differences in overall survival were quantified using Cox regression models to calculate hazard ratios (HR).

**Results:**

Across both cohorts, OCS patients were significantly older at diagnosis compared to all other histotypes (median age at diagnosis 69 and 67 in Scottish and SEER cohorts) and demonstrated the shortest survival time upon univariable analysis. Within the Scottish cohort, 59.3% and 16.9% of OCS patients presented with FIGO stage III and IV disease, respectively; this was significantly higher than in EnOC, CCOC or MOC (P<0.0001 for all), but lower than in HGSOC (P=0.004). Multivariable analysis accounting for other prognostic factors identified OCS as independently associated with significantly shorter survival time compared to HGSOC, EnOC, LGSOC and MOC in both the Scottish (multivariable HR vs OCS: HGSOC 0.45, EnOC 0.39, LGSOC 0.26, MOC 0.43) and SEER cohorts (multivariable HR vs OCS: HGSOC 0.59, EnOC 0.34, LGSOC 0.30, MOC 0.81). Within the SEER cohort, OCS also demonstrated shorter survival compared to CCOC (multivariable HR 0.63, 95% CI 0.58-0.68), but this was not replicated within the Scottish cohort (multivariable HR for CCOC: 1.05, 95% CI 0.74-1.51). Within early-stage disease specifically (FIGO I-II or SEER localized stage), OCS was associated with the poorest survival of all histotypes across both cohorts. In the context of late-stage disease (FIGO III-IV or SEER distant stage), OCS, MOC and CCOC represented the histotypes with poorest survival.

**Conclusion:**

OCS is a unique ovarian cancer type that affects older women and is associated with exceptionally poor outcome, even when diagnosed at earlier stage. New therapeutic options are urgently required to improve outcomes.

## Introduction

Ovarian carcinosarcoma (OCS) is an uncommon form of ovarian cancer, accounting for approximately 3% of diagnoses, and is distinguished by the presence of both carcinomatous and sarcomatous malignant cell populations (1–3). This biphasic histology led to the hypothesis that OCS may represent collisions of two separately originating tumors; however, the consensus has shifted over the last decade to recognize OCS as metaplastic carcinomas, with the sarcomatous population formed through complete epithelial-to-mesenchymal transition (1, 4). The unique history of OCS has resulted in its exclusion from many pan-histotype studies of ovarian carcinoma, leading to a paucity of research on OCS when compared to other uncommon histotypes (2).

Several studies have examined retrospective cohorts of OCS cases to identify factors associated with patient outcomes (5–11). These studies report a median survival time of 12-24 months across the broader OCS patient population. Earlier FIGO stage at diagnosis and achievement of complete macroscopic resection are both associated with more favorable prognosis, but recurrence and mortality rates appear high even in patients diagnosed with early-stage disease (11–13).

As most OCS cases have carcinomatous components of high grade serous type, some have conceptualized OCS as a rare variant of high grade serous ovarian carcinoma (HGSOC), the most common ovarian cancer histotype (2). However, a significant proportion have carcinomatous components of endometrioid type (3, 11), and limited comparisons of OCS and HGSOC have suggested significant differences in the behavior of these two histotypes (11, 13). Compared to HGSOC, OCS demonstrates greater levels of intrinsic chemoresistance (objective response rate between 30-60%) and is associated with an overall poorer prognosis (11, 14).

While efforts at characterizing the clinical behavior of OCS have improved our understanding of prognostic factors within OCS patients, and limited comparisons have been made against HGSOC (11, 15), there has been little comparison of OCS versus other ovarian carcinoma histotypes. Here, we compare OCS against all major epithelial ovarian carcinoma histotypes using two independent cohorts to improve our understanding of the clinical behavior of these uncommon tumors.

## Methods

### Scottish ovarian cancer patient cohort

**Figure 1:**
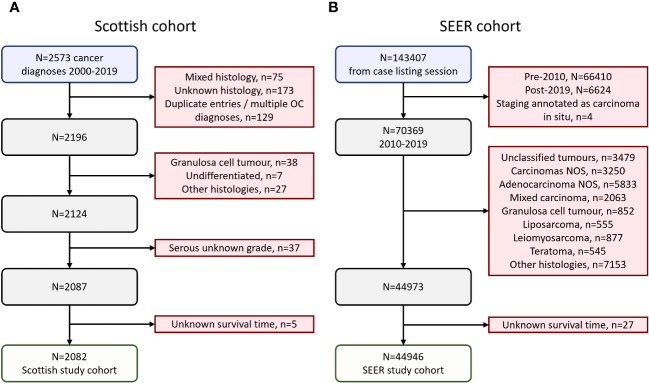
Flow diagrams of cohort identification. **(A)** Scottish ovarian cancer patient cohort. **(B)** SEER ovarian cancer patient cohort.

A cohort of ovarian cancer (ovarian, fallopian tube or primary peritoneal cancer) patients was identified using the Edinburgh Ovarian Cancer Database (16), wherein the diagnostic, treatment and outcome details of pathologically-confirmed ovarian cancer cases treated at the Edinburgh Cancer Centre (tertiary oncology centre for South-East Scotland) are prospectively recorded as part of routine care (16). Between 2000-2019, 2573 ovarian cancer diagnoses were documented, of which 2124 were carcinomas of serous (HGSOC or LGSOC), mucinous, carcinosarcoma, endometrioid or clear cell histology (**Figure 1A**). Older cases documented as poorly differentiated serous carcinoma and moderately differentiated serous carcinoma were included alongside contemporary diagnoses of HGSOC. Similarly, well differentiated serous carcinomas were included alongside contemporary diagnoses of LGSOC. Serous cases of unknown grade were excluded (n=37). 5 further cases were excluded due to unknown survival time, leaving a Scottish study cohort of 2082 cases (**Figure 1A**). Formal pathology review was not performed for the present study; however, 77% of cases recently underwent pathology review as part of tumour molecular profiling studies (4, 11, 17–26) or represented contemporary diagnoses (2010 onwards).

Institutional review board approval for the Scottish cohort was received from the South East Scotland Cancer Information Research Governance Committee (Caldicott guardian reference CG/DF/E164, study reference CIR21087).

### SEER ovarian cancer patient cohort

A cohort of ovarian cancer patients from the publicly available US Surveillance, Epidemiology, and End Results (SEER) program was identified using SEERstat version 8.4.2 (**Figure 1B**). 143407 cases of ovarian (C56.9), fallopian tube (C57.0) or peritoneal cancers (C48.0, C48.1, C48.2, C48.8) were retrieved in a case listing session (November 2022 SEER incidence research data: 2000-2020, 17 registries; selected for malignant behavior and primary site listed as C48.0, C48.1, C48.2, C48.8, C56.9 or C57.0). These cases were extracted, and the following exclusion criteria applied: diagnosis prior to 2010 (n=66410) or after 2019 (n=6624), carcinoma *in situ* (n=4), unspecified histology (n=12562), mixed histologies (n=2063), granulosa cell tumors (n=852), liposarcomas (n=555), leiomyosarcomas (n=877), teratomas (n=545), and other histologies beyond serous, endometrioid, clear cell, mucinous and carcinosarcoma (n=7153). A further 27 cases were excluded due to unknown survival time, leaving a SEER study cohort of 44946 cases (**Figure 1B**).

Stage was defined using combined SEER summary stage 2004+ data, identifying cases with localized-, regional-, or distant-stage disease. ICD-0-3 morphology codes were used to categories the SEER cohort into the following histotypes: endometrioid (ICD.O.3 8380, 8381, 8382, 8383 or 8570), mucinous (ICD.O.3 8470, 8471, 8472, 8480, 8481), clear cell (ICD.O.3 8310, 8313, 8443, 8444), carcinosarcoma (ICD.O.3 8575, 8950, 8951, 8980, 8981) and serous (ICD.O.3 8441, 8460, 8461, 8462). Serous cases annotated as well differentiated, grade 1 or low grade were classified as LGSOC; all other serious cases were included as HGSOC.

### Statistical analysis

All statistical analyses were performed using R version 4.2.2 within R Studio 2022.07.2 + 576. Comparisons of categorical variables were made using the Chi-squared test. Comparisons of continuous variables were made using the Mann-Whitney U test. For the Scottish cohort, overall survival was calculated from date of pathologically confirmed diagnosis. Cox proportional hazards regression models were used to compare survival across groups. Within the Scottish cohort, multivariable analysis accounted for age at diagnosis, FIGO stage at diagnosis, diagnosis period (5-year intervals) and residual disease status following first-line debulking surgery. For the SEER cohort, multivariable analysis accounted for disease stage, patient age and diagnosis period (5-year intervals). Results are visualized using the Kaplan-Meier method and survival differences are presented as hazard ratios (HRs) with respective 95% confidence intervals (CIs). The reverse Kaplan-Meier method was used to calculate median follow-up time. Statistical significance was defined as P<0.05.

## Results

### Scottish cohort characteristics

The Scottish cohort comprised 2082 patients with a pathologically-confirmed ovarian, fallopian tube or primary peritoneal cancer diagnosed between 2000-2019 (**Figure 1A**). 63 cases (3.0%) were OCS (**Table 1**). 1376 (66.1%), 231 (11.1%), 185 (8.9%), 146 (7.0%) and 81 (3.9%) were HGSOC, EnOC, CCOC, MOC and LGSOC, respectively, broadly reflecting previously reported histotype distributions in unselected ovarian carcinoma cohorts (4). The majority of cases presented with advanced stage disease (50.6% FIGO III, 972 of 1920 evaluable cases; 19.1% FIGO IV 366 of 1920). The median follow-up time across the cohort was 7.2 years; the survival event rate was 65.6% (**Table 1**).

**Table 1:** Characteristics of the Scottish ovarian cancer patient cohort.

		Overall		OCS		HGSOC		EnOC		CCOC		MOC		LGSOC	
		n	%	n	%	n	%	n	%	n	%	n	%	n	%
Cohort	N	2082		63		1376		231		185		146		81	
Age	Median	64	IQR 55-72	69	IQR 63-76	67	IQR 58-74	60	IQR 50-68	60	IQR 52-69	53	IQR 41-65	60	IQR 43-68
FIGO stage	I	365	19.0%	8	13.6%	59	4.7%	107	49.8%	71	40.6%	107	76.4%	13	16.9%
	II	217	11.3%	6	10.2%	74	5.9%	56	26.0%	52	29.7%	17	12.1%	12	15.6%
	III	972	50.6%	35	59.3%	810	64.6%	38	17.7%	34	19.4%	13	9.3%	42	54.5%
	IV	366	19.1%	10	16.9%	311	24.8%	14	6.5%	18	10.3%	3	2.1%	10	13.0%
	NA	162	–	4	–	122	–	16	–	10	–	6	–	4	–
Residual disease status	CMR	873	45.9%	29	48.3%	396	31.6%	166	76.9%	126	75.4%	119	89.5%	37	51.4%
	macroRD	1028	54.1%	31	51.7%	857	68.4%	50	23.1%	41	24.6%	14	10.5%	35	48.6%
	NA	181	–	3	–	123	–	15	–	18	–	13	–	9	–
ECOG performance status	0	372	26.7%	12	24.0%	215	22.7%	48	37.2%	49	38.6%	33	57.9%	15	32.6%
	1	604	43.4%	18	36.0%	434	45.8%	56	43.4%	53	41.7%	16	28.1%	27	58.7%
	2	287	20.6%	9	18.0%	232	24.5%	18	14.0%	19	15.0%	5	8.8%	4	8.7%
	3-4	130	9.3%	11	22.0%	103	10.9%	7	5.4%	6	4.7%	3	5.3%	0	0.0%
	NA	689	–	13	–	392	–	102	–	58	–	89	–	35	–
Progression status	Progressed	1276	61.3%	49	77.8%	1004	73.0%	64	27.7%	93	50.3%	26	17.8%	40	49.4%
	Stable	806	38.7%	14	22.2%	372	27.0%	167	72.3%	92	49.7%	120	82.2%	41	50.6%
Vital status	Alive	716	34.4%	7	11.1%	340	24.7%	146	63.2%	75	40.5%	107	73.3%	41	50.6%
	Deceased	1366	65.6%	56	88.9%	1036	75.3%	85	36.8%	110	48.1%	39	26.7%	40	49.4%
Follow-up	Median	7.2 years		8.4 years		7.0 years		7.9 years		6.4 years		7.5 years		8.5 years	

OCS, ovarian carcinosarcoma; HGSOC, high grade serous ovarian carcinoma; EnOC, endometrioid ovarian carcinoma; CCOC, clear cell ovarian carcinoma; MOC, mucinous ovarian carcinoma; LGSOC, low grade serous ovarian carcinoma; CMR, complete macroscopic resection after primary cytoreduction; macroRD, macroscopic residual disease after primary cytoreduction; ECOG, Eastern Cooperative Oncology Group.

“-”, not calculated.

### Comparison of histotypes with carcinosarcoma

**Figure 2:**
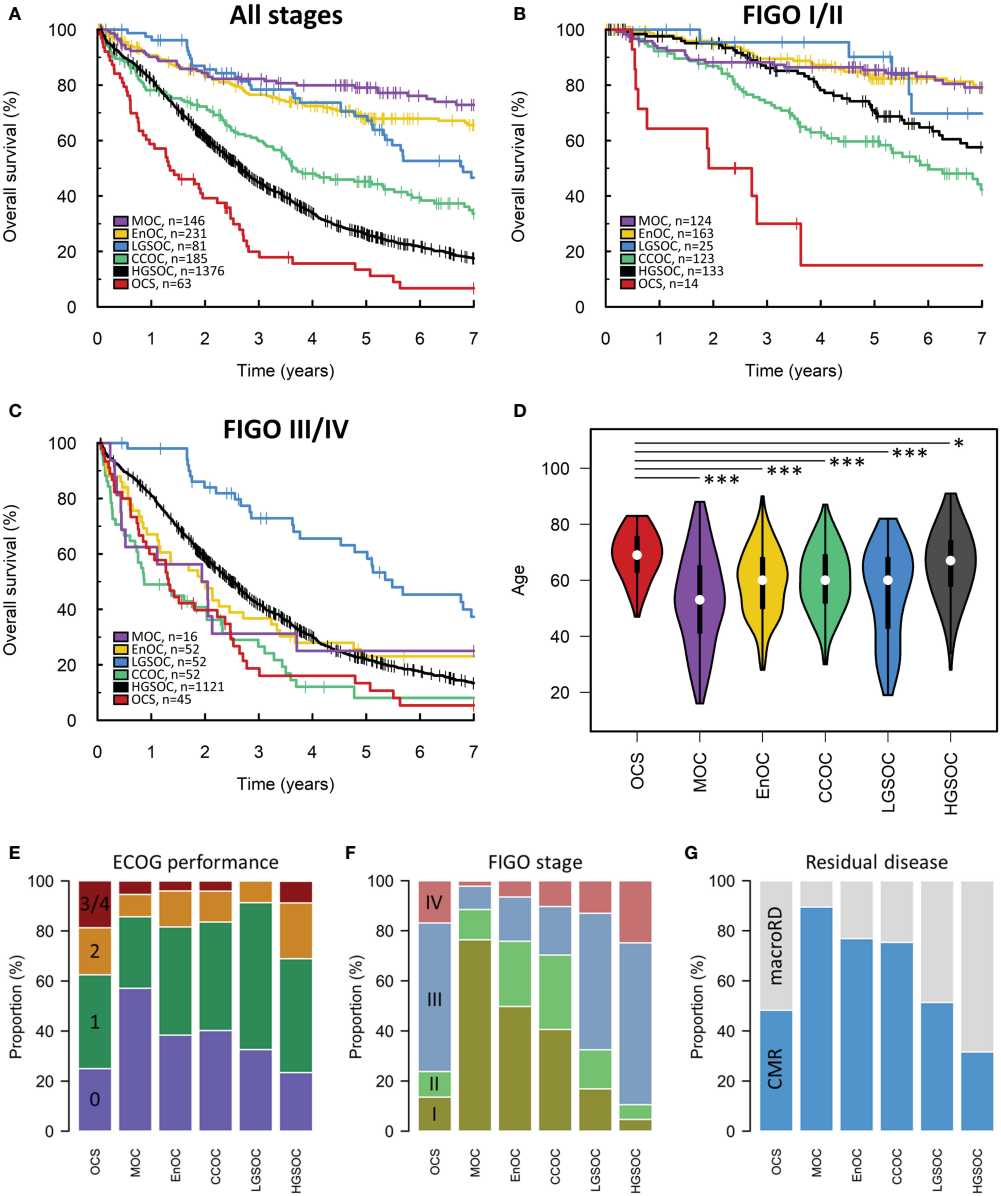
Scottish ovarian cancer patient cohort. **(A)** Survival of patient cohort according to histotype. **(B)** Survival analysis of early-stage patients (FIGO I-II). **(C)** Survival of late-stage patients (FIGO III-IV). **(D)** Age at diagnosis of patients according to histotype. *denotes P<0.05, ***denotes P<0.0001. **(E)** ECOG performance status according to histotype. Chi-squared P-values for comparison of ECOG PS (≤1 vs ≥2) in histotypes against OCS: MOC P=0.0125, EnOC P=0.0145, CCOC P=0.0056, LGSOC P=0.0023, HGSOC P=0.4322. **(F)** FIGO stage at diagnosis. Chi-squared P-values for comparison of stage distribution in histotypes against OCS: MOC P<0.0001, EnOC P<0.0001, CCOC P<0.0001, LGSOC P=0.7459, HGSOC P=0.0140. **(G)** Frequency of achieving complete macroscopic resection (CMR) versus macroscopic residual disease (macroRD) according to histotype. OCS, ovarian carcinosarcoma; HGSOC, high grade serous ovarian carcinoma; EnOC, endometrioid ovarian carcinoma; CCOC, clear cell ovarian carcinoma; MOC, mucinous ovarian carcinoma; LGSOC, low grade serous ovarian carcinoma.

The median survival time of OCS patients was 17 months (**Figure 2A**). Univariable survival analysis identified OCS as the histotype associated with the poorest survival outcomes (HR vs OCS: HGSOC 0.55, 95% CI 0.42-0.72; CCOC 0.36, 95% CI 0.26-0.50; LGSOC 0.21, 95% CI 0.14-0.32; EnOC 0.15, 95% CI 0.11-0.21; MOC 0.10, 95% CI 0.07-0.15) (**Figure 2A**). However, clinicopathological features varied significantly between histotypes; patients with OCS were significantly older at diagnosis compared to all other histotypes (median 69 years in OCS vs 67, 60, 60, 53 and 60 in HGSOC, EnOC, CCOC, MOC and LGSOC, respectively) (**Figure 2D**) with corresponding higher Eastern Cooperative Oncology Group (ECOG) Performance Status scores (**Figure 2E**). Stage distribution was also markedly different between histotypes: OCS cases had a higher frequency of early-stage (FIGO I/II) diagnosis compared to HGSOC (23.7%, 14/59 evaluable OCS vs 10.1%, 133/1254 evaluable HGSOC; P=0.004), but a higher frequency of advanced stage (FIGO III/IV) at diagnosis compared to MOC (P<0.0001), EnOC (P<0.0001) and CCOC (P<0.0001) (**Figure 2F**). Corresponding differences in frequency of achieving complete macroscopic resection (CMR, zero residual disease/R0) at first-line surgery were also apparent (**Figure 2G**). Together, these data highlight the need for multivariable analysis.

**Figure 3:**
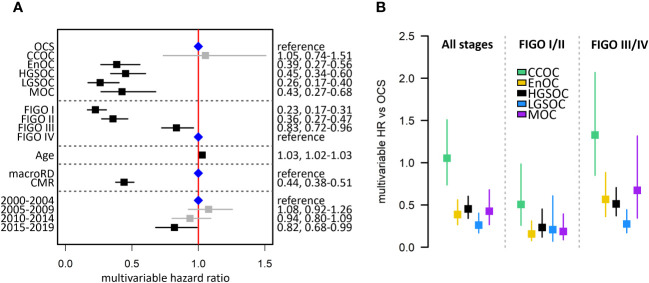
Summary of multivariable survival analysis model in the Scottish study cohort. **(A)** Multivariable model across all stages. Blue points denote the reference groups for each level; black points denote statistically significant differences; grey denotes estimates not statistically significantly different from the reference population. **(B)** Summary of multivariable hazard ratios across the whole cohort, early-stage-specific analysis and late-stage-specific analysis. OCS, ovarian carcinosarcoma; HGSOC, high grade serous ovarian carcinoma; EnOC, endometrioid ovarian carcinoma; CCOC, clear cell ovarian carcinoma; MOC, mucinous ovarian carcinoma; LGSOC, low grade serous ovarian carcinoma; CMR, complete macroscopic resection after primary cytoreduction; macroRD, macroscopic residual disease after primary cytoreduction; HR, hazard ratio.

Multivariable analysis of survival accounting for patient age, stage at diagnosis, residual disease status and diagnosis period identified OCS as a histotype associated with significantly poorer outcome compared to HGSOC (multivariable HR [mHR] for HGSOC vs OCS 0.45, 95% CI 0.34-0.60), EnOC (mHR vs OCS: 0.39, 95% CI 0.27-0.56), MOC (mHR vs OCS 0.43, 95% CI 0.27-0.68) and LGSOC (mHR vs OCS: 0.26, 95% CI 0.17-0.40) (**Figure 3A**). There was no significant difference in survival of CCOC patients vs OCS patients in this multivariable analysis (mHR for CCOC vs OCS: 1.05, 95% CI 0.74-1.51).

### Outcome in early- and late-stage disease

Survival analysis of patients diagnosed at early-stage (FIGO I-II) identified OCS as a patient group with markedly poor outcome (**Figure 2B**). OCS was associated with significantly shorter survival than all other histotypes in a multivariable analysis accounting for age, stage (I vs II), RD status and diagnosis period; this included significantly shorter survival in early-stage OCS versus early-stage CCOC (mHR for CCOC vs OCS: 0.51, 95% CI 0.26-0.98) (**Figure 2B**).

A corresponding analysis of advanced stage patients (FIGO III/IV) showed late-stage OCS was associated with shorter survival compared to late-stage HGSOC, EnOC and LGSOC (**Figures 2C**, **3B**); differences between late-stage OCS and MOC (mHR vs OCS: 0.67, 95% CI 0.34-1.32) and CCOC (HR vs OCS: 1.33, 95% CI 0.85-2.07) were not statistically significant.

### SEER cohort characteristics

A second cohort of 44946 ovarian cancer patients was identified from the SEER database (**Figure 1B**). 2030 (4.5%), 30706 (68.3%), 5336 (11.9%), 3088 (6.9%), 2846 (6.3%) and 940 (2.1%) cases were OCS, HGSOC, EnOC, CCOC, MOC and LGSOC, respectively (**Table 2**). The median follow-up time for the SEER cohort was 5.6 years, with a survival event rate of 48.9%.

**Table 2:** Characteristics of the SEER ovarian cancer patient cohort.

		Overall		OCS		HGSOC		EnOC		CCOC		MOC		LGSOC	
		n	%	n	%	n	%	n	%	n	%	n	%	n	%
Cohort	N	44946		2030		30706		5336		3088		2846		940	
Age	Median	63	IQR 54-72	67	IQR 59-75	65	IQR 56-73	55	IQR 47-65	57	IQR 50-65	55	IQR 43-65	58	IQR 45-67
SEER Stage	Localized	7411	16.8%	108	5.4%	2196	7.3%	2297	43.5%	1125	36.9%	1498	53.9%	187	20.2%
	Regional	10432	23.6%	461	23.2%	5770	19.1%	2124	40.3%	1161	38.1%	665	23.9%	251	27.1%
	Distant	26330	59.6%	1414	71.3%	22192	73.6%	856	16.2%	763	25.0%	618	22.2%	487	52.6%
	NA	773	–	47	–	548	–	59	–	39	–	65	–	15	–
Vital status	Alive	22980	51.1%	602	29.7%	17356	56.5%	4292	80.4%	2071	67.1%	1943	68.3%	722	76.8%
	Deceased	21966	48.9%	1428	70.3%	13350	43.5%	1044	19.6%	1017	32.9%	903	31.7%	218	23.2%
Follow-up	Median	5.6 years		5.8 years		5.6 years		5.6 years		5.3 years		5.6 years		4.6 years	

OCS, ovarian carcinosarcoma; HGSOC, high grade serous ovarian carcinoma; EnOC, endometrioid ovarian carcinoma; CCOC, clear cell ovarian carcinoma; MOC, mucinous ovarian carcinoma; LGSOC, low grade serous ovarian carcinoma.

“-”, not calculated.

### Comparison of histotypes in the SEER cohort

**Figure 4:**
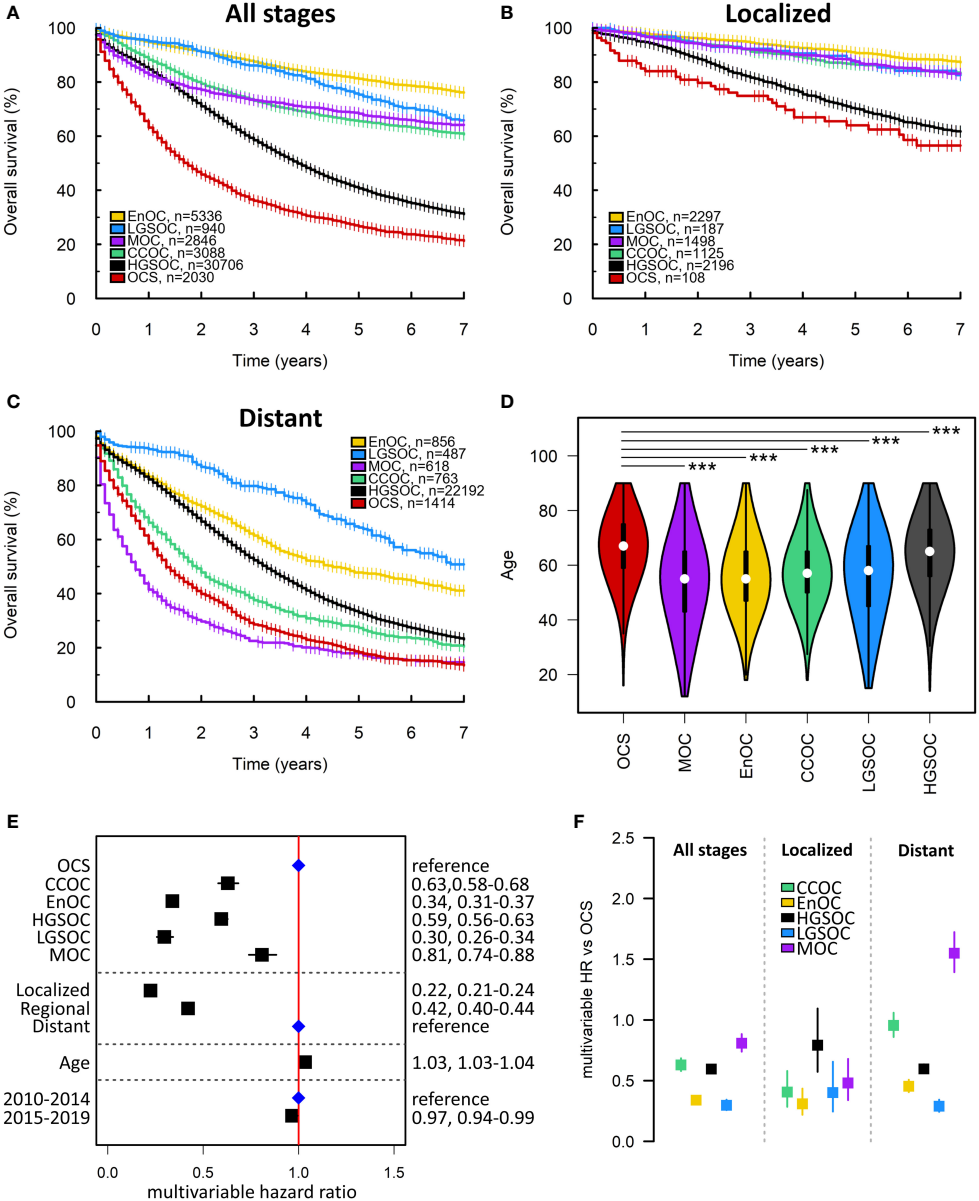
SEER ovarian cancer patient cohort. **(A)** Survival of the whole SEER cohort (Early, Regional and Distant stage) according to histotype. **(B)** Survival analysis of early-stage patients (Localized disease). **(C)** Survival of late-stage patients (Distant disease). **(D)** Age at diagnosis of patients according to histotype. ***denotes P<0.0001. **(E)** Multivariable survival analysis across all stages. Blue points denote the reference groups for each level; black points denote statistically significant differences. **(F)** Summary of multivariable hazard ratios across the whole cohort, early-stage-specific analysis and late-stage-specific analysis. OCS, ovarian carcinosarcoma. HGSOC, high grade serous ovarian carcinoma; EnOC, endometrioid ovarian carcinoma; CCOC, clear cell ovarian carcinoma; MOC, mucinous ovarian carcinoma; LGSOC, low grade serous ovarian carcinoma; HR, hazard ratio.

The median survival time of OCS patients within the SEER cohort was 21 months (**Figures 4A–C**). Within the SEER cohort, OCS demonstrated the shortest survival time upon univariable analysis (**Figure 4A**) and was associated with significantly older age at diagnosis compared to other histotypes (median 67, 65, 55, 57, 55 and 58 in OCS, HGSOC, EnOC, CCOC, MOC and LGSOC, respectively; P<0.0001 for all comparisons against OCS) (**Figure 4D**). Multivariable analysis identified significantly shorter survival in OCS patients compared to all other histotypes (mHR vs OCS: HGSOC 0.59, 95% CI 0.56-0.63; EnOC 0.34, 95% CI 0.31-0.37; CCOC 0.63, 95% CI 0.58-0.68; MOC 0.81, 95% CI 0.74-0.88; LGSOC 0.30, 95% CI 0.26-0.34) (**Figure 4E**).

Within the earliest SEER disease stage (Localized disease), OCS demonstrated the shortest survival of all histotypes (**Figure 3B**), though the difference between OCS and HGSOC did not reach statistical significance (mHR for HGSOC vs OCS: 0.79, 95% CI 0.57-1.09) (**Figure 4F**). Within the most advanced SEER stage (Distant disease), OCS was associated with poorer survival than HGSOC (mHR for HGSOC vs OCS: 0.60, 95% CI 0.56-0.63), EnOC (mHR for EnOC vs OCS: 0.45, 95% CI 0.41-0.51) and LGSOC (mHR for LGSOC vs OCS: 0.29, 95% CI 0.24-0.34) (**Figure 3B**). The outcome of late-stage OCS and CCOC was similar (mHR for CCOC vs OCS: 0.95, 95% CI 0.86-1.06), while late-stage MOC demonstrated the poorest outcome of all histotypes at late-stage (mHR for MOC vs OCS: 1.56, 95% CI 1.40-1.73) (**Figure 4F**).

## Discussion

OCS is now recognized as a histotype of ovarian carcinoma, but has received relatively little research attention to date (2). Limited comparisons have been made against HGSOC (11, 13), the most common histotype, but there is a paucity of data comparing these unusual tumors against the spectrum of major ovarian cancer histotypes. Here, we utilize two independent cohorts of ovarian cancer patients to comprehensively characterize the clinical behavior of OCS.

Our findings highlight several distinct features of OCS compared to other ovarian carcinoma histotypes. Firstly, OCS presents in women at an older age compared to other histotypes: the median age at diagnosis in OCS was 69 years in the Scottish cohort and 67 years in the SEER cohort, and this was statistically significantly older than all other histotypes across both cohorts. Within HGSOC, the other histotype with a median diagnosis age of over 60 years, copy number gain of *CCNE1* has been associated with older age at diagnosis (17). OCS have recently been reported to commonly demonstrate *CCNE1* gain (4), and their older age at diagnosis may be linked to the frequency of this defect; however, direct comparison of *CCNE1* status and age of OCS diagnosis has not been reported.

OCS also appears to have a distinct stage distribution; the majority of OCS present at FIGO stage III-IV – unlike MOC, EnOC and CCOC – but around 25% are FIGO stage I/II at diagnosis, and this is significantly more than in HGSOC. As OCS frequently present with advanced stage disease, many patients undergo neoadjuvant chemotherapy prior to cytoreductive surgery; this approach is widely considered safe and effective for HGSOC (27, 28), but neoadjuvant chemotherapy versus primary debulking surgery has not been specifically compared for OCS. Given reports of higher levels of intrinsic chemoresistance in OCS (objective response rate 25-60%) (11–13), neoadjuvant chemotherapy may feasibly represent a less effective management strategy. Indeed, neoadjuvant chemotherapy is not the preferred approach for other histotypes with high levels of intrinsic chemoresistance (29). However, challenges in identifying OCS on diagnostic biopsies – where the sarcomatous component may not be sampled, leading to a diagnosis of more common carcinoma hisotypes – may interfere with the ability to tailor early first-line management decisions for OCS patients.

Univariable analysis identified OCS as the histotype associated with poorest survival across both cohorts. Within the Scottish cohort, multivariable analysis demonstrated that this was independent of other prognostic factors for comparisons of OCS against all other histotypes, with the exception of CCOC. The poorer outcome of OCS compared to HGSOC, EnOC, LGSOC and MOC was confirmed in the SEER cohort; this cohort also identified OCS as having significantly shorter survival than CCOC. The difference in comparisons with CCOC between cohorts may be underpinned by greater statistical power in the SEER cohort, though less detailed clinical annotation prevented inclusion of residual disease status in the SEER cohort model, likely contributing to this discrepancy. Together, these data suggest that the overall OCS population represents the highest risk histotype across ovarian carcinomas.

In an analysis specifically of earlier stage patients (FIGO I-II) in the Scottish cohort, OCS was associated with markedly shorter survival than all other histotypes, including CCOC. These findings were replicated when investigating SEER cohort patients with localized disease, though the comparison with HGSOC was not statistically significant (mHR for localized HGSOC vs localized OCS 0.80, 95% CI 0.58-1.11). This discrepancy may be due to the difference in staging between cohort; SEER localized stage equates to the very earliest FIGO stages (IA, IB and stage I not otherwise specified). These data have important implications for decisions around omission of chemotherapy for early-stage disease. Many ovarian cancer cases diagnosed at the earliest stages do not require chemotherapy (28); however, the aggressive nature of early-stage OCS suggests that chemotherapy omission may not be advisable for this group. Similarly, fertility-sparing surgery may not be feasible in this context, though most OCS patients present after reproductive age. In late-stage disease (FIGO III-IV), the Scottish cohort demonstrated that OCS was associated with significantly shorter survival compared to HGSOC, LGSOC and EnOC, but was not associated with significantly poorer outcome than CCOC or MOC. These findings were confirmed in the SEER cohort, where distant stage CCOC demonstrated similar survival to distant stage OCS, and late-stage MOC demonstrated the worst survival of all histotypes in this context. Together, these stage-specific analyses highlight OCS as highly aggressive even when diagnosed at early-stage, while in the context of late-stage disease, OCS, CCOC and MOC represent the histotypes with poorest survival. This is consistent with reports highlighting CCOC and MOC as highly chemoresistant malignancies with exceptionally poor prognosis when diagnosed at advanced stage (2). While OCS is most commonly diagnosed at advanced stage, late-stage diagnosis of CCOC and MOC is relatively uncommon, underscoring treatment of late-stage OCS as a major clinical challenge.

Major strengths of this study include the detailed clinical annotation available for the Scottish cohort, extensive follow-up time and the utilization of multivariable analysis to assess associations of histotype with outcome independent of other prognostic factors. The use of two independent cohorts from distinct geographical locations is also a notable strength; SEER is a pan-cancer database curated across a large number of centres in the US, while the Edinburgh Ovarian Cancer Database is a disease-specific resource curated centrally at a single site. A limitation of the present study is that all cases did not undergo centralized pathology review, though over 75% of the Scottish cohort has either undergone pathology review as part of recent molecular profiling studies or represented contemporary diagnoses (2010 onwards), limiting the potential for histotype misclassification. As it was not possible to perform pathology review of any cases in the SEER cohort, we utilized only recent diagnoses from the SEER database (2010-2019) to minimize potential histotype misclassification. Though we were able to include a relatively large number of cases with uncommon histotypes - and the number of these cases exceeded that in many reported cohorts of these less common diagnoses - power was still limited for some analyses. In particular, the number of advanced stage MOC and early-stage OCS or LGSOC cases was modest, though the large effect sizes detected between analyses of these groups bolstered power.

Our findings highlight the urgent need for additional treatment options for OCS patients. Molecular profiling studies have the potential to identify targeted approaches that may improve OCS patient survival; however, relatively few OCS samples have undergone genomic, transcriptomic or other molecular characterization to date. Limited available data suggest a paucity of targetable oncogenic driver mutations from the genomes of OCS tumors (4, 30), with *TP53* mutation representing one of the few recurrent molecular events. A proportion of OCS demonstrate genomic evidence of homologous recombination repair deficiency (31), and these cases may be expected to benefit from poly(ADP-ribose) polymerase (PARP) inhibitors. Case reports of OCS patients deriving clinical benefit from PARP inhibition are available in the literature, but this evidence base is extremely limited (32, 33). The frequency of germline or somatic *BRCA1*/*2* mutation is poorly characterized in OCS; case reports of *BRCA1*/*2*-mutant OCS are available, but current data from OCS cohorts suggest the frequency is low (0/12 in (4) and 0/13 in (34)). There is also a lack of data quantifying the extent of homologous recombination deficiency with robustly established techniques due to a lack of whole genome sequencing (35).

Recent data suggest that the sarcomatous compartment of OCS is less well engaged by the host anti-tumour immune response compared to the carcinomatous component (4); immunotherapeutic drugs may therefore represent agents worthy of investigation in the hope of reinvigorating the anti-tumour immune response. In particular, immune checkpoint inhibitors targeting PD1, PDL1 and CTLA4 are of interest. Case reports of responses to such inhibitors in OCS patients provide anecdotal evidence of their potential utility in the wider population (36, 37). However, as with other candidate targeted approaches, there is a marked absence of trial data at any phase. Overexpression of HER2 and VEGF in some OCS has suggested trastuzumab and anti-angiogenics as further potential treatment strategies for investigation, alongside inhibitors of mTOR (38). Recently established preclinical models of OCS have identified eribulin as a candidate therapeutic strategy targeting epithelial-to-mesenchymal transition in OCS (39), and we eagerly await the results from initial clinical evaluations of this strategy (NCT05619913). The relative rarity of OCS is likely to hinder progress of histotype-specific trials for this tumour type; international collaborative efforts have led to successful disease-specific trials in other uncommon ovarian cancer types (40), and it is likely that similar international collaboration will be required to drive advances in the standard of care for OCS patients.

As with other uncommon ovarian cancer histotypes, a multidisciplinary approach is key for determining optimal management for individuals with OCS.

## Conclusion

Together, our findings identify OCS as an exceptionally aggressive histotype of ovarian carcinoma. OCS patients represent an older patient group that are frequently diagnosed at advanced stage. Despite its aggressive behavior, OCS is a relatively under-researched tumour type, hindering progress toward new treatment options which are urgently required to improve outcomes.

## Data availability statement

The Scottish dataset presented in this article is not readily available because sharing of patient data is only possible within the constraints of our local ethics framework, which means line-by-line data cannot be shared without seeking an associated ethical approval. Such approval can be sought via contact with the corresponding author (robb.hollis@ed.ac.uk). Data from the SEER database can be accessed through the SEER program website. 

## Ethics statement

Institutional review board approval for the Scottish cohort was received from the South East Scotland Cancer Information Research Governance Committee (Caldicott guardian reference CG/DF/E164, study reference CIR21087). The studies were conducted in accordance with the local legislation and institutional requirements. The need for informed consent for every participant was waived due to the retrospective nature of the study.

## Author contributions

IM: Data curation, Formal analysis, Visualization, Methodology, Writing – original draft. JMP: Data curation, Writing – review and editing. EB: Data curation, Writing – review and editing. NG: Resources, Writing – review and editing. KCC: Resources, Writing – review and editing. CSH: Resources, Writing – review and editing. RLH: Conceptualization, Data curation, Formal analysis, Methodology, Visualization, Writing – original draft.
